# Bradykinin B2 Receptor Contributes to Inflammatory Responses in Human Endothelial Cells by the Transactivation of the Fibroblast Growth Factor Receptor FGFR-1

**DOI:** 10.3390/ijms19092638

**Published:** 2018-09-06

**Authors:** Erika Terzuoli, Federico Corti, Ginevra Nannelli, Antonio Giachetti, Sandra Donnini, Marina Ziche

**Affiliations:** 1 Dept. Life Sciences, University of Siena, Via A. Moro 2, 53100 Siena, Italy; terzuoli8@unisi.it (E.T.); nannelli5@student.unisi.it (G.N.); okkamm@gmail.com (A.G.); 2 Yale Cardiovascular Research Center, Section of Cardiovascular Medicine, Dept. of Internal Medicine, Yale University School of Medicine, New Haven, CT 06520, USA; federico.corti@yale.edu

**Keywords:** bradykinin, B2R antagonist, endothelial cells, FGFR-1

## Abstract

Elevated levels of bradykinin (BK) and fibroblast growth factor-2 (FGF-2) have been implicated in the pathogenesis of inflammatory and angiogenic disorders. In angiogenesis, both stimuli induce a pro-inflammatory signature in endothelial cells, activating an autocrine/paracrine amplification loop that sustains the neovascularization process. Here we investigated the contribution of the FGF-2 pathway in the BK-mediated human endothelial cell permeability and migration, and the role of the B2 receptor (B2R) of BK in this cross-talk. BK (1 µM) upregulated the FGF-2 expression and promoted the FGF-2 signaling, both in human umbilical vein endothelial cells (HUVEC) and in retinal capillary endothelial cells (HREC) by the activation of Fibroblast growth factor receptor-1 (FGFR-1) and its downstream signaling (fibroblast growth factor receptor substrate: FRSα, extracellular signal–regulated kinases1/2: ERK1/2, and signal transducer and activator of transcription 3: STAT3 phosphorylation). FGFR-1 phosphorylation triggered by BK was c-Src mediated and independent from FGF-2 upregulation. Either HUVEC and HREC exposed to BK showed increased permeability, disassembly of adherens and tight-junction, and increased cell migration. B2R blockade by the selective antagonist, fasitibant, significantly inhibited FGF-2/FGFR-1 signaling, and in turn, BK-mediated endothelial cell permeability and migration. Similarly, the FGFR-1 inhibitor, SU5402, and the knock-down of the receptor prevented the BK/B2R inflammatory response in endothelial cells. In conclusion, this work demonstrates the existence of a BK/B2R/FGFR-1/FGF-2 axis in endothelial cells that might be implicated in propagation of angiogenic/inflammatory responses. A B2R blockade, by abolishing the initial BK stimulus, strongly attenuated FGFR-1-driven cell permeability and migration.

## 1. Introduction

Inflammation and angiogenesis are closely integrated processes regulating a number of physiological and pathological settings, including wound healing, rheumatoid arthritis, diabetic retinopathy, arteriosclerosis, and cancer [1,2,3]. Fibroblast growth factor-2 (FGF-2) exerts a key role in the cross-talk between angiogenesis and inflammation by interacting with various surface molecules, including tyrosine kinase receptors Fibroblast growth factor receptor 1 to 4 (FGFR-1 to FGFR-4), heparan-sulfate proteoglycans, integrins and syndecans [4]. In endothelial cells (EC), through the FGFR-1 activation, FGF-2 promotes the inflammatory response during the angiogenic process by inducing vasoactive effects, such as vasodilation and vascular permeability [5,6,7]. Further, the FGF-2/FGFR-1 signaling pathway promotes the expression of a wide variety of inflammation-related genes in EC, which have a pivotal role in the neovascularization [8,9], and, in turn, several inflammatory mediators trigger FGF-2 release from EC [10,11]. In a previous report, we showed that prostaglandin E2 (PGE2) induces angiogenesis by an autocrine FGF-2 mobilization from the EC extracellular matrix, resulting in FGFR-1 signaling activation [12,13]. Of note, FGF-2 itself also upregulates its expression in EC, and that of other growth factors, including vascular endothelial growth factor (VEGF) [14,15,16]. Thus, the EC activation by FGF-2 appears to be a concerted action between an autocrine loop and that of other factors originating in a paracrine modality from inflammatory cells, including prostanoids, cytokines, and other chemokines [17,18].

Two G-protein-coupled receptors, BK receptor 1 and 2 (B1R and B2R) transduce BK signals to the effector molecules mentioned above [19]. Activation of these receptors elicits pro-angiogenic responses in EC, as well as important changes of their tone (vasodilatation), permeability, and the enhanced recruitment of inflammatory cells [17,20,21]. Recently, in the oxygen induced retinopathy (OIR) model, in mice, we demonstrated that the BK/B2R signaling plays a pathogenic role in retinal neovascularization, and that its effects correlate with FGF-2 upregulation in retinal vessels [22]. Here we investigated the contribution of the FGF-2/FGFR-1 pathway to BK/B2R-mediated human endothelial cell permeability and migration in two EC lines, human umbilical vein endothelial cells (HUVEC) and human retinal capillary endothelial cells (HREC). Our data demonstrates that BK transactivated the FGF-2/FGFR-1 signaling in both HUVEC and HREC cells, which was implicated in cell permeability and migration. B2R blockade, by abolishing the initial BK stimulus, strongly attenuated FGFR-1-driven inflammatory responses.

## 2. Results

### 2.1. BK/B2R System Transactivates FGFR-1 and Drives Its Downstream Signaling in EC

**Figure 1:**
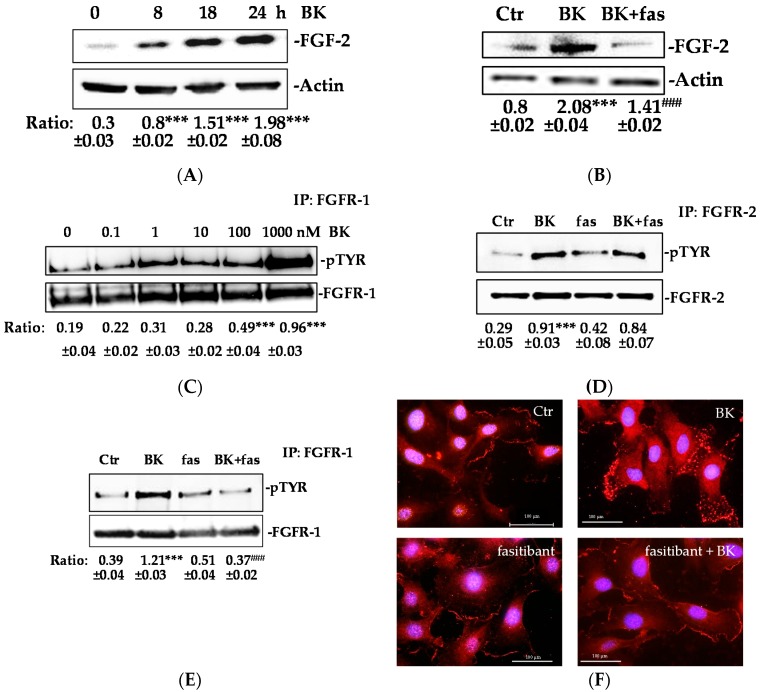
BK/B2R transactivates FGFR-1 and mediates its internalization. (**A**) HUVEC were treated with BK (1 μM) for 8, 18, and 24 h, and FGF-2 expression was evaluated using western blot analysis. Results were normalized to actin. Quantification was expressed as an arbitrary density unit (ADU). The results presented are representative of three independent experiments (*n* = 3) with similar results. (**B**) HUVEC were treated with fasitibant (fas, 1 µM, 30 min), then stimulated with BK (1 μM) for 24 h, and FGF-2 expression was evaluated using western blot analysis. Results were normalized to actin. (**C**) HUVEC were treated with BK (0.1–1000 nM, 10 min), FGFR-1 was immunoprecipitated (IP), and its activation was investigated by anti-pTYR antibody. Results were normalized to FGFR-1. (**D**,**E**) HUVEC were treated with fasitibant (fas, 1 µM, 30 min), then stimulated with 1 μM BK (10 min), FGFR-2 and FGFR-1 were immunoprecipitated (IP), and its activation was investigated by anti-pTYR antibody. Results were normalized to FGFR-2 and FGFR-1, respectively. *** *p* < 0.001 vs. Ctr; ^###^ *p* < 0.001 vs. BK treated cells. Ctr (control, 0.1% FBS). (**F**) Immunofluorescence analysis of FGFR-1 localization in endothelial cells in the control condition (Ctr, 0.1% FBS) and in the presence of BK (1 μM, 10 min) alone or in combination with fasitibant (1 μM). Magnification, 100×, scale bar = 100 μm.

In order to investigate whether the BK/B2R promotes the FGF-2/FGFR-1 signaling activation, we determined the FGF-2 expression in HUVEC exposed to BK. BK increased the FGF-2 expression time-dependently (max at 24 h) (Figure 1A), whereas pre-treatment of HUVEC with fasitibant (fas), a B2R selective antagonist, produced a consistent reduction of the FGF-2 expression (Figure 1B). Further, we examined whether BK might stimulate FGFR-1 phosphorylation in EC. FGFR-1, and less frequently FGFR-2, are expressed by EC, while FGFR-3 and FGFR-4 are not [23]. The treatment with BK (0.1–1000 nM) induced rapid FGFR-1 phosphorylation with a maximal activity observed at 1 μM (Figure 1C). A concentration of 1 μM BK also induced FGFR-2 phosphorylation (3-fold increase over basal, Figure 1D). Inversely, pre-treatment of HUVEC with fasitibant (fas) produced a significant decrement of FGFR-1 phosphorylation (without affecting FGFR-2 activity) (Figure 1D,E). These results indicate the specificity of the BK/B2R signaling pathway in the activation of the FGF2/FGFR-1 system (Figure 1B–E).

We also studied the perinuclear translocation of FGFR-1 in response to BK, a known mechanism linked to tyrosine kinase receptor activation [12].

In agreement with the FGFR-1 phosphorylation, Figure 1F shows a different pattern of FGFR-1 staining in ECs exposed for 10 min to BK (upper panel, on the right) suggesting that BK may promote the FGFR-1 translocation from the membrane/cytoplasm to the perinuclear area, while fasitibant blocked the receptor internalization (Figure 1F, bottom panel, on the right).

**Figure 2:**
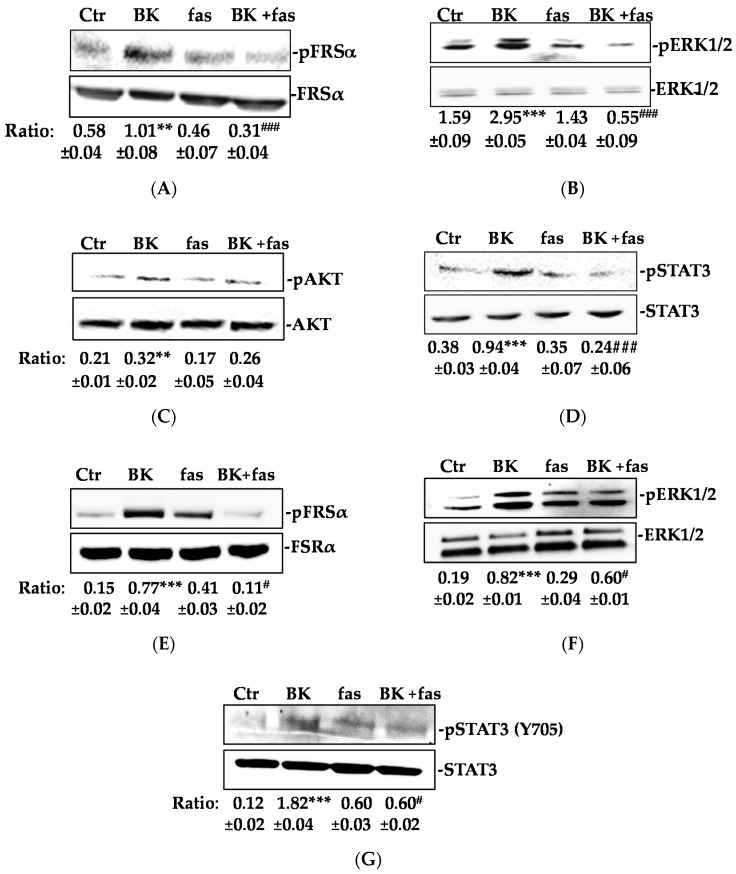
BK/B2R activates FGFR-1 signaling. (**A**) FRSα, (**B**) ERK1/2, (**C**) AKT, and (**D**) STAT3 phosphorylation were evaluated using western blot analysis in HUVEC treated with fasitibant (fas, 1 μM, 30 min), then stimulated with BK (1 μM) for 15 min. (**E**) FRSα, (**F**) ERK1/2, and (**G**) STAT3 phosphorylation were evaluated using western blot analysis in HREC treated with fasitibant (fas, 1 μM, 30 min), then stimulated with BK (1 μM) for 15 min. Results were normalized to FRSα, ERK1/2, AKT, and STAT3, respectively. The results presented are representative of three independent experiments (*n* = 3) with similar results. Quantification was expressed as an arbitrary density unit (ADU). ** *p* < 0.01; *** *p* < 0.001 vs. Ctr; ^#^ *p* < 0.05; ^###^ *p* < 0.001 vs. BK treated cells.

Next, we investigated the effect of BK/B2R signaling on the second messengers downstream to FGFR-1. BK (1 µM, 15 min) induced the phosphorylation of fibroblast growth factor receptor substrate (FRSα), extracellular signal–regulated kinases1/2 (ERK1/2), Protein Kinase B (AKT), and signal transducer and activator of transcription 3 (STAT3) (Figure 2A–D) in HUVEC. Similar results were obtained in HREC (Figure 2E–G). Fasitibant (fas) inhibited the phosphorylation of all the above second messengers, except AKT (Figure 2A–G).

**Figure 3:**
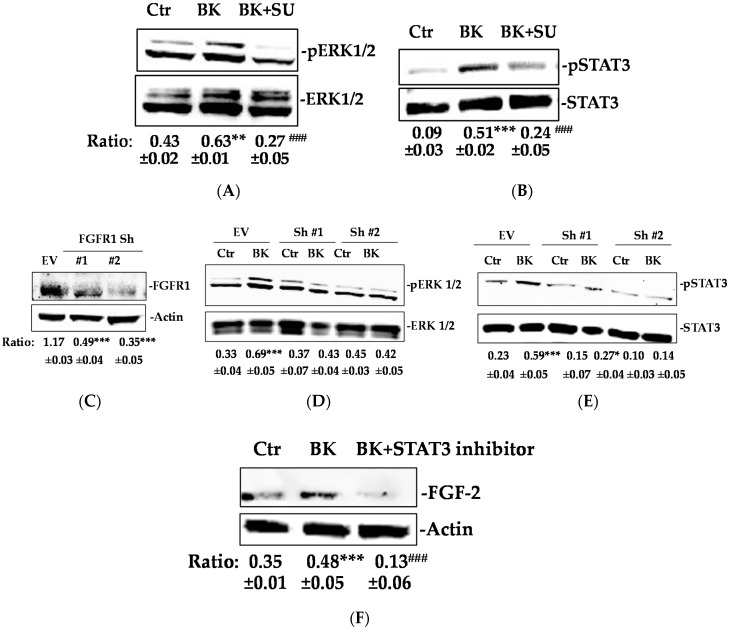
BK-mediated ERK1/2-STAT3/FGF-2 signaling activation requires FGFR-1. (**A**) ERK1/2 and (**B**) STAT3 phosphorylation were evaluated using western blot analysis in HUVEC treated with SU5402 (1 μM, 30 min), then stimulated with BK (1 μM) for 15 min. Results were normalized to ERK1/2 and STAT3, respectively. (**C**) FGFR-1 expression evaluated using western blot analysis in HUVEC transfected with two different shRNA for FGFR-1 knock-down (Sh#1 and Sh#2). EV = empty vector. (**D**,**E**) Western blot analysis for ERK1/2 and STAT3 phosphorylation in HUVEC transfected with Sh#1 and Sh#2 and stimulated with BK (1 μM) for 15 min. (**F**) FGF-2 expression was evaluated in HUVEC treated with STAT3 inhibitor (10 μM, 30 min) and then stimulated with BK (1 μM) for 24 h. Actin was used as a loading control. The results presented are representative of three independent experiments (*n* = 3) with similar results. Quantification was expressed as an arbitrary density unit (ADU). ** *p* < 0.01; *** *p* < 0.001 vs. Ctr; ^###^ *p* < 0.001 vs. BK treated cells.

BK/B2R signaling is reported to directly promote ERK1/2 and STAT3 activation [24,25]. However, we found that exposure to SU5402, a FGFR-1 inhibitor, before the BK challenge, strongly reduced the ERK1/2 and STAT3 activation, suggesting that FGFR-1 lay downstream to BK in promoting EC activation (Figure 3A,B). Similarly, knocking-down FGFR-1 in HUVEC inhibited BK activation of ERK1/2 and STAT3 (Figure 3C–E). Also, a STAT3 inhibitor pretreatment reduced the BK-induced FGF-2 expression, indicating that the FGF-2 upregulation occurred downstream through the FGFR-1/STAT3 signaling pathway activation (Figure 3F).

### 2.2. Phosphorylation of FGFR-1 by BK Requires the Activation of c-Src

**Figure 4:**
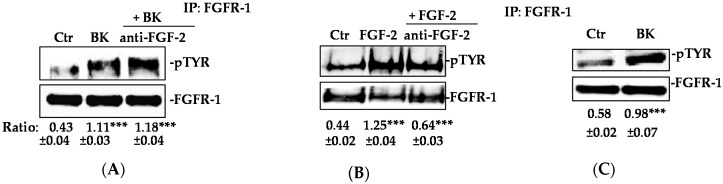
BK phosphorylates FGFR-1 despite the absence of FGF-2. (**A**,**B**) HUVEC were treated with anti-FGF-2 neutralizing antibody (6 μg/mL), then stimulated with FGF-2 (20 ng/mL), as a positive control or BK (1 μM) for 10 min. FGFR-1 was immunoprecipitated (IP), and its activation was investigated by anti-pTYR antibody. Results were normalized to FGFR-1. *** *p* < 0.001 vs. Ctr (control, 0.1% FBS). (**C**) Murine EC was isolated from FGF-2^−/−^ mice were stimulated with BK (1 μM) for 10 min. FGFR-1 was immunoprecipitated (IP), and its activation was investigated by an anti-pTYR antibody. Results were normalized to FGFR-1. The results presented are representative of three independent experiments (*n* = 3) with similar results. *** *p* < 0.001 vs. Ctr (control, 0.1% FBS).

To investigate the mechanism whereby BK stimulated the phosphorylation of FGFR-1, we designed experiments in which FGF-2 was either ablated (knockout) or its signal transduction was impaired through the application of a non-permeant specific neutralizing antibody to EC. Both maneuvers failed to influence the FGFR-1 phosphorylation elicited by the BK exposure (Figure 4A,B), leading to the conclusion that BK activates the FGFR-1 pathway independently from the extant FGF-2.

**Figure 5:**
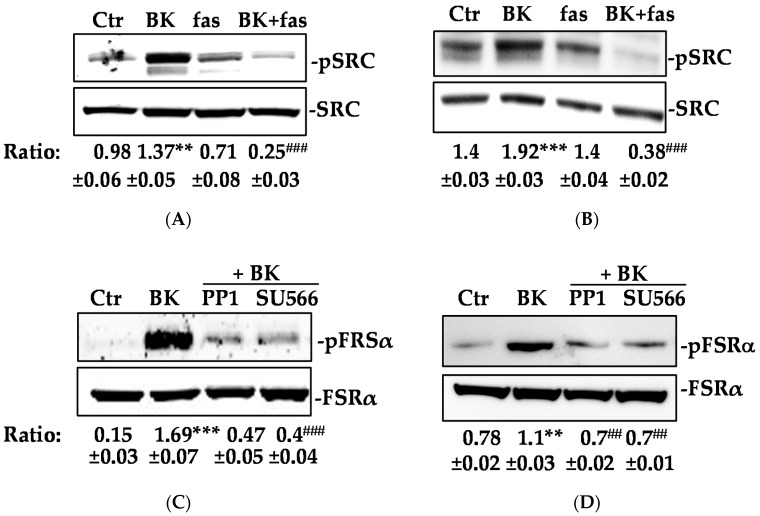

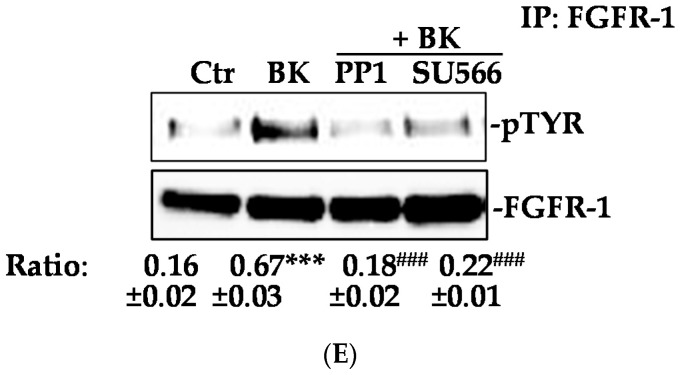
c-Src mediates FGFR-1 phosphorylation induced by BK/B2R system. (**A**) HUVEC and (**B**) HREC were treated with fasitibant (fas, 1 μM), then stimulated with BK (1 μM) for 15 min, and c-SRC phosphorylation was measured using western blot analysis. Results were normalized to SRC. FRSα phosphorylation was measured using western blot analysis in (**C**) HUVEC and (**D**) HREC treated with PP1 (500 nM), or SU566 (10 μM) (Src inhibitors) for 30 min, and then stimulated with BK (1 μM) for 15 min. Results were normalized to FRSα. (**E**) HUVEC were treated with PP1 (500 nM), or SU566 (10 μM) as above, and then stimulated with BK (1 μM) for 10 min. FGFR-1 was immunoprecipitated (IP), and its activation was investigated by an anti-pTYR antibody. Results were normalized to FGFR-1. The gels shown are representative of three experiments obtained with similar results. ** *p* < 0.01, *** *p* < 0.001 vs. Ctr (control, 0.1% FBS). ^##^ *p* < 0.01, ^###^ *p* < 0.001 vs. BK treated cells.

Given the observed irrelevancy of the extracellular FGFR-1 domain in the downstream signal propagation activated by BK/B2R system, we focused on the cell cytosol, particularly on c-Src, a kinase recently shown to serve as a signaling mediator both downstream and upstream of the epidermal growth factor receptor activation [26]. BK application to EC (HUVEC and HREC) promoted a robust increase over basal in c-Src phosphorylation via B2R, an effect sensitive to fas blockade (5-fold decrease over BK treated cells; Figure 5A,B). Inhibition of FGFR-1 and FRSα phosphorylation mediated by BK through the known c-Src inhibitors PP1 and SU566, provided evidence that FGFR-1 lay upstream of c-Src (Figure 5C–E). We conclude that BK activated FGFR-1 through a c-Src-dependent mechanism that appeared to be independent to the FGF-2-mediated receptor activation.

### 2.3. BK Induces Endothelial Permeability and Migration through FGFR-1 Activation

**Figure 6:**
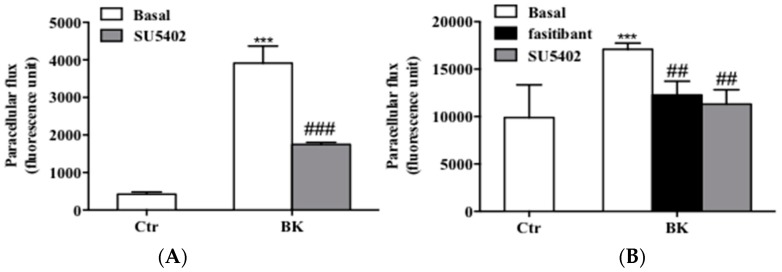

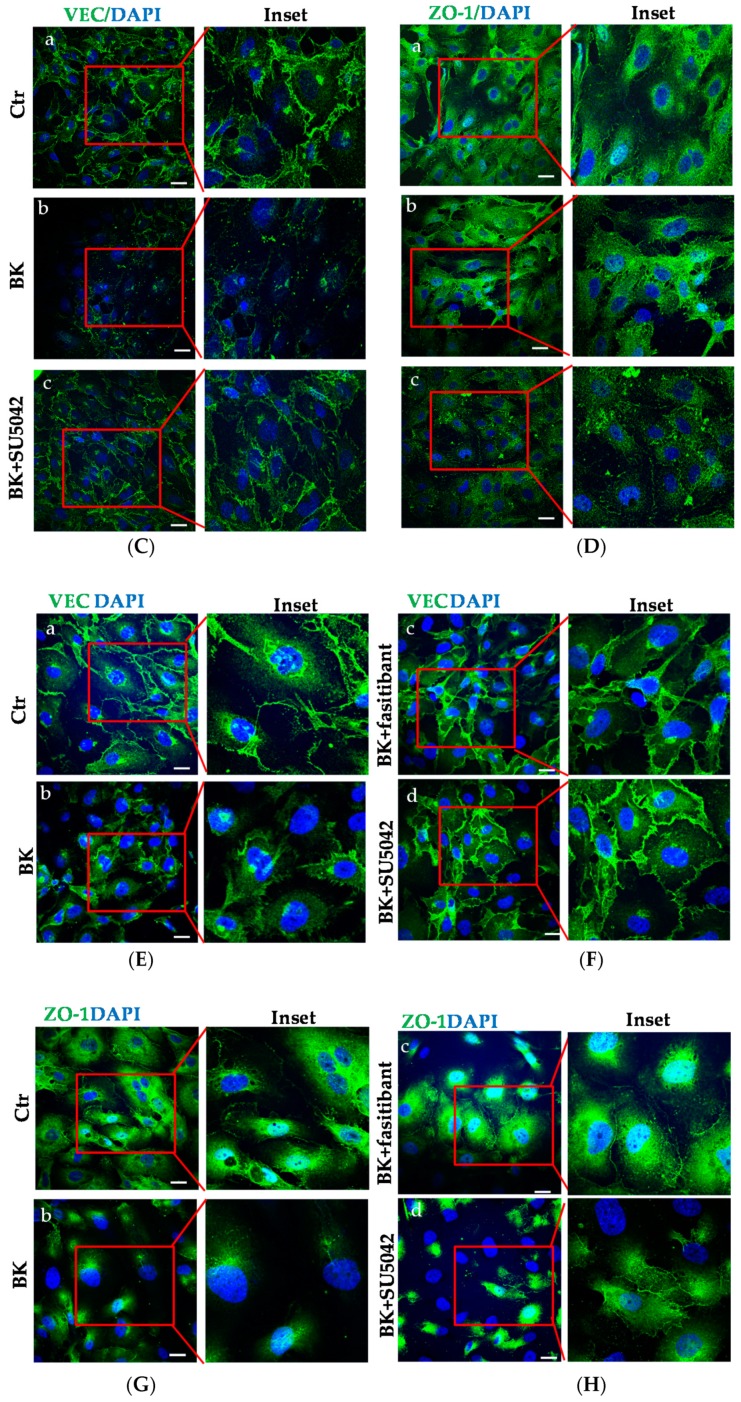
BK/B2R system promotes changes of permeability, endothelial junctions and migration via FGFR-1. (**A**) Permeability in HUVEC and (**B**) HREC monolayers were detected as a passage of fluorescence-conjugated FITC-Dextran from upper to lower compartments (numbers represent mean 6 ± SEM of three experiments run in triplicate; *n* = 3). Fasitibant (1 µM) and SU5402 (1 µM) prevent the enhanced permeability, *** *p* < 0.001 vs. Ctr cells, ^##^ *p* < 0.01 ^###^ *p* < 0.001 vs. BK-treated cells. (**C**,**D**) Confocal analysis of VEC and ZO-1 expression (magnification 63×) evaluated by immunofluorescence analysis in HUVEC treated with 0.1% FBS in panel a, BK (1 µM) in panel b, and SU5402 + BK in panel c. (**E**,**F**) Confocal analysis of VEC and (**G**,**H**) ZO-1 expression (magnification 63×) evaluated by immunofluorescence analysis in HREC treated with 0.1% FBS in panel a, BK (1 µM) in panel b, BK + fasitibant (10 µM) in panel c, and SU5402 + BK in panel d. Bar = 20 µm. VEC and ZO-1 were stained in green and DAPI (blue) was used to counterstain the nuclei. Boxed areas are shown in detail in the inset.

As previously demonstrated, BK produces a significant increase of endothelial permeability, a common histopathological marker of inflammation, measured as paracellular flux of fluorescent conjugated dextran [17]. In EC, BK increased paracellular flux. Co-treatment of EC with fasitibant (1 µM) or SU5402 (1 µM) abolished the BK-induced paracellular flux increase, restoring the flux to the control level (Figure 6A,B).

In a condition of in vitro confluence, cells regulate permeability through the expression of cell-type-specific transmembrane adhesion proteins, such as vascular endothelial-cadherin (VEC), at adherens junctions, and zonula occludens-1 (ZO-1), at tight junctions. Consistent with its permeability effects, BK drastically reduced the typical pattern of fluorescence localization of either VEC (Figures 6C,E panel b vs. panel a) or ZO-1 (Figure 6D,G panel b vs. panel a) at the cell–cell contacts. Fasitibant and SU5402 prevented the cytoskeletal organization disruption of both adhesion proteins (Figure 6C,D, panels c vs. panel b; Figure 6E–H panel c or d vs. panel b). We previously demonstrated that fasitibant recovers the cytoskeletal organization of either VEC and ZO-1 in HUVEC [17]. These observations clearly indicate that activation of FGFR-1 signaling mediates BK-induced permeability.

**Figure 7:**
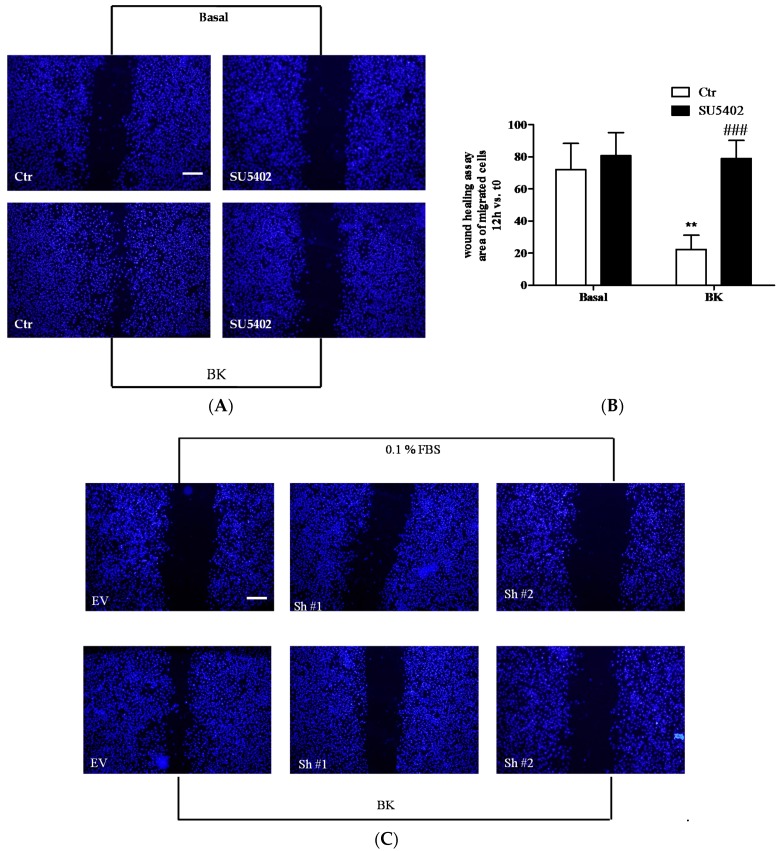

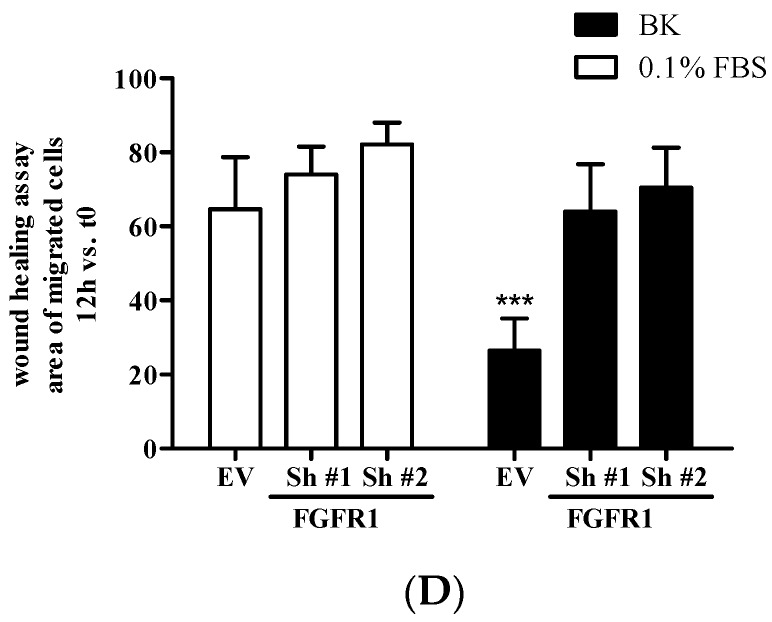
(**A**,**C**) Scratch wound healing assay on HUVEC treated with: in (**A**), 0.1% FBS (Basal, Ctr), BK (1 µM) (BK, Ctr), SU5402 (1 µM), SU5402 + BK; in (**C**), 0.1% FBS (EV), BK (1 μM) (BK, EV), FGFR-1 knock down (0.1% FBS, Sh#1 and Sh#2), FGFR-1 knock-down + BK (BK, Sh#1 and Sh#2). (**B**,**D**) Quantification of cell migration was reported as the area of migrated cells. Figure B is the quantification of figure A and figure D is the quantification of figure C. ** *p* < 0.01 vs. Ctr cells; ^###^ *p* < 0.001 vs. BK-treated cells. Numbers represent mean 3 ± SEM of three experiments run in triplicate. Bar = 100 µm.

We also evaluated the contribution of FGFR-1 activation in the pro-angiogenic effect of BK by studying endothelial cell migration. In EC, BK induced migration while SU5402 co-incubated with BK, and the knock-down of FGFR-1 reduced its effects (Figure 7A,C and Figure 7B,D for quantification), indicating that FGFR-1 was involved in the pro-angiogenic effect of BK in EC.

## 3. Discussion

In this study, we demonstrate that stimulation of FGF-2/FGFR-1 signaling in endothelial cells contributes to BK/B2R-induced permeability changes and migration. These findings suggest that FGF-2 and BK signaling might orchestrate the pathogenesis of vascular disorders through induction of inflammatory and proangiogenic changes in the vascular endothelium.

The half-life of BK is regulated locally and is propagated through stimulation of other local signaling [27]. Recently, in a model of OIR, in mice, we demonstrated that BK/B2R is involved in the pathological retinal neovascularization and that this effect correlated with upregulation of FGF-2 in retinal vessels [22]. A limited number of studies suggest the existence of a functional link between the two systems in several inflammatory/angiogenic disorders [28,29,30]. Although FGF-2 can trigger neovessel formation per se, the major finding of this study concerns the BK ability to transactivate FGFR-1 signaling in endothelial cells, as evidenced by its phosphorylation and translocation from the plasma membrane to cytosol, and, in turn, by the phosphorylation of second messengers downstream to FGFR-1, as FRSα, ERK1/2, and STAT3. All events occur within minutes from BK addition to EC, suggesting that the peptide functions as an initial trigger for a robust pro-angiogenic response. Further, the delayed upregulation of FGF-2 observed in EC challenged with BK indicate that the B2R signaling also functions as a trigger for an amplified pro-angiogenic/inflammatory response mediated by FGF-2 signaling. Fasitibant application to EC, a selective full antagonist of B2R, halted the FGFR-1 activation, therefore inhibiting the functional downstream sequelae of its activation. Of note, from several of our findings, in absence of BK, the antagonist appeared to affect the B2R signaling per se. Further experiments are needed to investigate this point. Interfering with the FGF-2/FGFR-1 system by a FGFR-1 blockade (inhibition of the receptor through the low selective SU5402 [31], or its knocking-down through shRNA) also inhibited BK activity on ERK1/2 and STAT3, and similarly, STAT3 inhibition suppressed BK activity on FGF-2 expression, indicating that the FGF-2/FGFR-1 system plays a functional role in the proangiogenic/proinflammatory effects of BK. It is notable that STAT3 is a key player in inflammation. The interactions of growth factors and cytokines with their membrane-bound receptors frequently trigger STAT activation [32,33,34]. FGFR-1 influences the STAT3 pathway, and in turn, impacts on pro-inflammatory and proangiogenic responses [32,33,34]. In EC exposed to BK, STAT3 appears instrumental for FGF-2 upregulation. Further, the endothelium, activated through B2R stimulation and by the FGFR-1 cascade products, exhibits the typical inflammatory/angiogenic phenotype, as shown by enhanced permeability and motility of endothelial cells.

**Figure 8:**
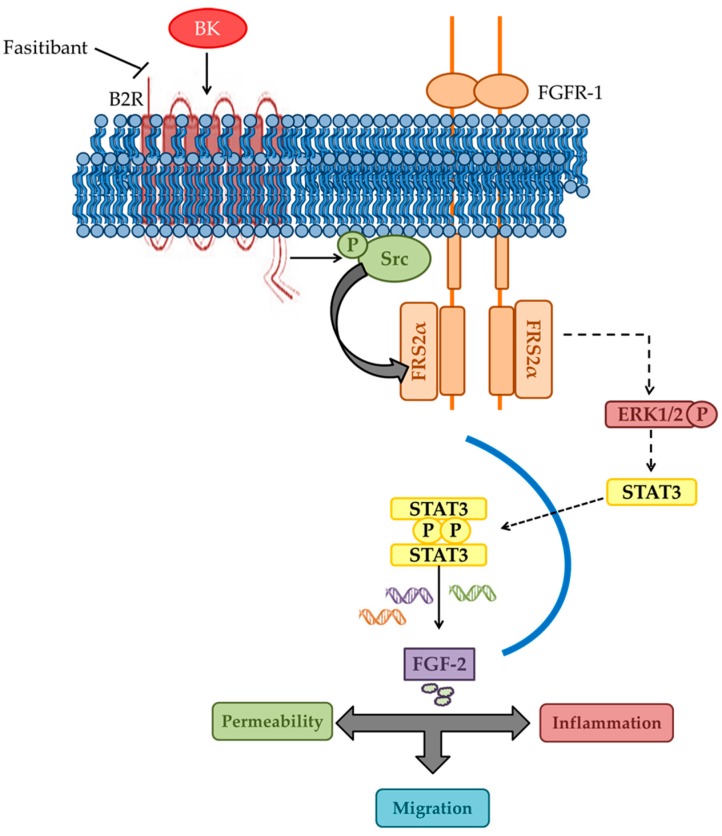
Schematic model of BK/B2R-FGF-2/FGFR-1 interaction in endothelial cells. The figure depicts the interaction between BK and FGF-2 signaling in endothelial cells. Dotted arrows indicate complex signaling pathways involving several second messengers.

In light of these results, we propose a model for the angiogenic/inflammatory switch in EC based on the FGFR-1 signaling stimulation and FGF-2 upregulation by BK (see Figure 8). Previously, we reported that FGFR-1 acts as a master switch in neovascularization induced by inflammatory mediators by initiating a positive autocrine/paracrine loop of FGF-2 synthesis and FGFR-1 activation [13]. In EC, BK appears to act as a primer of this switch by directly transactivating FGFR-1 signaling and FGF-2 expression. In conclusion, the functional mechanistic association between the BK and FGF-2 signaling pathways here described provides the basis for a defined targeting of molecules involved in microvascular disease initiation and progression. The blockade of the B2R by a selective antagonist might restrict the pathological angiogenesis, reducing the acute inflammatory and angiogenic responses of the vascular endothelium and the following amplification and propagation through the FGFR-1/FGF-2 pathway.

## 4. Materials and Methods

### 4.1. Cell Culture

Human retinal endothelial cells (HREC, passages 3–7) were from Innoprot, (Basque Country, Spain) and certified for expression of CD31/105, von WFVIII. Cells were grown in endothelial cell medium (ECM) (Innoprot, Basque Country) supplemented with 10% FBS and antibiotics (100 U/mL penicillin, 100 μg/mL streptomycin) on fibronectin-coated dishes. Cells between passage 3 to 7 were used in these experiments.

HUVEC were purchased from Lonza (Basel, Switzerland), grown in endothelial growth medium (EGM-2) (EBM-2, plus VEGF, R3-IGF-1, hEGF, hFGF, hydrocortisone, ascorbic acid, heparin, and GA-1000) (Clonetics, Cambrex Bio Science, Walkersville, MD, USA) and supplemented with 10% FBS antibiotics (100 U/mL penicillin, 100 μg/mL streptomycin) on gelatin-coated dishes. Cells were split 1:3 twice a week, and used until they reached passage 8. Both endothelial cell lines were cultured at 37 °C in 5% CO_2_. HUVEC FGFR-1 knockdown (Sh FGFR-1 #1 or Sh#2) and non-target shRNA (EV, empty vector) were cultured in presence of 10 µg/mL puromicin. A HUVEC cell line was transfected with shRNA plasmids by using Lipofectamine 3000 (Life Technologies, Monza, MB, Italy). Stably transfected clones were isolated with puromycine (10 µg/mL). Murine EC isolated from FGF-2^−/−^ mice were kindly provided by Paolo. Mignatti (NYU, New York, USA) and cultured as described [35].

### 4.2. FGFR-1 shRNA Transfection

Plasmids for FGFR1 knock-down were MISSIONTM shRNA pLKO.1-puro plasmids (with TCRN0000312516 (Sh#1) insert with sequence: 5’-CCGGGATGGCACCCGAGGCATTATTCTCGAGAATAATGCCTCGGGTGCCATCTTTTTG-3’ or with TRCN0000327645 (Sh#2) insert with sequence 5’-CCGGAGTGGCTTATTAATTCCGATACTCGAGTATCGGAATTAATAAGCCACTTTTTTG-3′) were obtained from Sigma Aldrich (Milan, MI, Italy). psPA × 2 packaging plasmid (12260) and pMDG.2 envelope plasmid (12259) were obtained from Addgene (Cambridge, MA, USA). All the plasmids were sequence verified.

To generate FGFR-1 knock-down cells (Sh#1 or Sh#2 cells), 1 × 10^6^ HEK293 cells (Life Technologies) were transfected with 2.25 μg of PA × 2 packaging plasmid, 0.75 μg of PMD2G envelope plasmid, and 3 μg of pLKO.1 hairpin vector utilizing 12 μL of Lipofectamine 2000 on 10 cm plates. Polyclonal populations of transduced cells were generated by infection with 1 MOI (multiplicity of infectious units) of lentiviral particles. At 3 days post infection, cells were selected with 10 µg/mL puromicin (Gibco, Thermo Fisher Scientific, Waltham, MA, USA) for 1 week.

### 4.3. Immunofluorescence Analysis

3 × 10^4^ cells were seeded on 1-cm-round glass coverslips. After 24 h, cells were washed and treated with the indicated stimuli. Cells were fixed in 4% paraformaldehyde/PBS with Ca^2+^ and Mg^2+^. Unspecific binding sites were blocked in 3% bovine serum albumin (BSA) with FGFR-1 without previous cell permeabilization in 0.25% Tween 20 in PBS for 10 min. Then, cells were incubated with a monoclonal mouse anti-FGFR-1 antibody (Merk Millipore, Darmstadt, Germany) diluted 1:25, a rabbit monoclonal anti-VE-cadherin (Cell Signaling, Milan, Italy) diluted 1:400, and a polyclonal rabbit anti-ZO-1 (ThermoFisher Scientific, Paisley, UK) diluted 1:50 in 0.5% BSA in PBS for 18 h at 4 °C. Cells were then washed and incubated with Alexafluor 555 or 488 anti-rabbit (ThermoFisher Scientific, Paisley, UK) diluted 1:200 in PBS with 0.5% BSA for 1 h. The cells were counterstained with DAPI for 20 min (Sigma Aldrich). Coverslips were mounted in fluoromount (Sigma Aldrich) and pictures of stained cells were taken using a confocal microscope, Leica SP5 confocal (Leica Microsystems, Milan, MI, Italy), at 63× magnification.

### 4.4. Western Blot Assay

Cells (3 × 10^5^/6 cm plate) were treated with BK (1 μM) with or without the pre-treatment with fasitibant (1 µM), SU5402 (1 μM), PP1 (500 nM) or SU566 (Src inhibitors) (10 μM), or STAT3 inhibitor VII (10 µM) for 30 min. pFRSα (Tyr196, #3864 Cell Signaling), FRSα (#MAB4069 R&D system), pERK1/2 (#4370 Cell Signaling), ERK1/2 (#9102 Cell Signaling), pSTAT3 (#9134 Cell Signaling), STAT3 (#9139 Cell Signaling), pAKT (#9271 Cell Signaling), AKT (#2920 Cell Signaling), pTYR (#9411 Cell Signaling), p-SRC (#6943 Cell Signaling), SRC (#2108 Cell Signaling), FGFR-1 (#9740 Cell Signaling), FGFR-2 (#3116 Cell Signaling), and FGF-2 (#05–118 Merk Millipore) expression were evaluated using western blot analysis [17]. Proteins (50 μg) from cell extracts were electrophoresed in SDS/4–12% polyacrylamide gels (Life Technologies). Proteins were then blotted onto activated nitrocellulose membranes, blocked with non-fat milk (Bio-Rad, Milan, MI, Italy), incubated overnight with the indicated antibodies, and antigen-antibody complexes were detected with enhanced chemiluminescence kit (Bio-Rad). Band intensity was measured using scanning densitometry.

### 4.5. Immunoprecipitation

Cells were stimulated with BK (0.1, 1, 10, 100, or 1000 nM) or with FGF-2 (20 ng/mL, R&D system) for 10 min. Where indicated, cells were pretreated with the anti-FGF-2 neutralizing antibody (6 μg/mL), PP1 (500 nM), or SU566 (Src inhibitors) (10 μM) or fasitibant (1 μM) for 30 min. Anti-FGFR-1 (#05-149 Merk Millipore) or FGFR-2 (#3116 Cell Signaling) were added to the precleared lysates (250 μg). Western blot was performed as previously described [17].

### 4.6. Wound Assay

Cells (1 × 10^5^ cells/well) were seeded into 24-well plates to reach confluence and then scraped to create a wound of ±1 mm width. SU5402 (1 μM) in the presence/absence of BK (1 μM, 12 h), where indicated, was added with the antimitotic ARA-C (2.5 µg/mL, Sigma Aldrich). Images of the wound in each well were acquired from 0 to 12 h under a phase contrast microscope, Nikon Eclipse TE 300 (Nikon, Tokyo, Japan), at 20× magnification. Results are expressed as the area of migrated cells, 12 h vs. time 0. After 12 h, the cells were stained with Hoechst 33342 (Life Technologies), and images were obtained as described above [17].

### 4.7. Permeability

Cells were seeded at 1 × 10^5^ on fibronectin-coated or gelatin-coated insert membranes (Corning, New York, NY, USA) with 0.4 μM diameter pores, and the inserts were placed in 12 multiwell plates. After 48 h, confluent monolayers were treated with SU5402 (1 μM) in the presence/absence of BK (10 μM) for 10 h. Then 3 kDa FITC-Dextran (10 μM) was added on top of cells, allowing the fluorescent molecules to pass through the cell monolayer toward the lower compartment. The extent of permeability was determined after 10 min by measuring the fluorescence in the medium present in the bottom of the well in a multiplate reader (SpectraFluor, Tecan, MI, Italy), at 485/535 nm, excitation/emission, respectively. Results are reported as relative fluorescence units (RFU) [17].

### 4.8. Materials and Reagents

Cell culture reagents—BK, SU566, ARA-C, DAPI, anti-β-Actin—were purchased from Sigma Aldrich (Merk Millipore). Fetal Bovine Serum (FBS) was from Innoprot (Basque Country). Anti-FGF-2 neutralizing antibody, STAT3 inhibitor VII, PP1, and SU5402 were purchased from Calbiochem (Merk Millipore, Darmstadt, Germany). FGF-2 was from R&D system. Fasitibant was kindly provided by Menarini Ricerche (Florence Italy). Anti-pTYR, anti-FGFR-1, anti-FGFR-2, anti-pFRSα, anti-pERK1/2, anti-ERK1/2, anti-pSTAT3, anti-STAT3, anti-pAKT, anti-AKT, anti-pSRC, and anti-SRC antibodies were from Cell Signaling (Milan, Italy). Anti-FRSα was from R&D system. Anti-FGF-2 was from Merk Millipore (Darmstadt, Germany).

### 4.9. Data Analysis and Statistical Procedures

Results are either representative or the average of at least three independent experiments done in triplicate. Statistical analysis was performed using a two way ANOVA test followed by a Bonferroni test (GraphPad). *p* < 0.05 was considered statistically significant.

## Abbreviations

ADU	Arbitrary Density Unit
BK	Bradykinin
BSA	Bovine serum albumin
B2R	BK receptor 2
fas	Fasitibant
FGF-2	Fibroblast growth factor-2
FGFR-1	FGF-receptor-1
HREC	Human retinal endothelial cells
NV	Neovessels
OIR	Oxygen induced retinopathy
PBS	Phosphate buffer saline
TYR	Tyrosine
VEC	Vascular endothelial cadherin
VEGF	Vascular endothelial growth factor
ZO-1	Zona occludens 1
